# Strategic Responses to Employee Well-Being Issues in VUCA (Volatile, Uncertain, Complex, and Ambiguous) Conditions: Expanding the JD-R (Job Demands–Resources) Model with Job Crafting

**DOI:** 10.3390/ijerph22010014

**Published:** 2024-12-26

**Authors:** Thomas Volderauer, Margit Raich, Antje Bierwisch, Harald Stummer, Oliver Som

**Affiliations:** 1 Institute for Management and Economics in Health Care, UMIT TIROL—Private University for Health Sciences and Health Technology, 6060 Hall in Tirol, Austria; thomas.volderauer@umit-tirol.at (T.V.); harald.stummer@umit-tirol.at (H.S.); 2 Vice-Rectorat for Research and Development, University College of Teacher Education Tyrol, 6010 Innsbruck, Austria; margit.raich@ph-tirol.ac.at; 3 Department of Innovation & Technology Management, Management Center Innsbruck, 6020 Innsbruck, Austria; antje.bierwisch@mci.edu; 4 Institute Health Management, IMC University of Applied Sciences Krems, 3500 Krems, Austria

**Keywords:** job demands resource model, occupational psychology, leadership, knowledge management, digital transformation, employee well-being, qualitative comparative case studies

## Abstract

Current work environments, driven by globalization, demographic changes, and digitalization, demand substantial adaptation, which leads to decreased employee well-being. While occupational psychology research has identified supportive mechanisms, it often lacks a deepened understanding of how interventions function. This study aims to analyze the impacts of VUCA contexts and leadership behavior on job crafting, focusing on white-collar workers. Our objective is to identify workplace configurations that safeguard employee health in dynamic settings. Utilizing the JD-R model augmented by job crafting as a theoretical framework, we conducted qualitative comparative case studies using the GABEK^®^ methodology to analyze and systematize data. This approach captures complex organizational interrelationships through sentence analysis, transforming subjective meanings into a comprehensive network and providing deeper insights into organizational dynamics. Research findings reveal that evolving work demands, notably from digital transformations, impact knowledge management, communication, and employee well-being. IT discrepancies and communication deficits intensify work complexity, underscoring the need for enhanced management frameworks. Proactive and adaptive leadership, along with job crafting, is crucial for protecting health and enhancing performance in fast-paced digital environments. These strategies promote structured decision-making and efficient knowledge management, fostering a resource-oriented culture that enhances employee well-being and overall effectiveness.

## 1. Introduction

In today’s rapidly evolving economic climate, marked by continual dynamism and change, organizations increasingly face a multitude of challenges. It is essential to align strategic objectives with employee needs to maintain long-term competitiveness. Agility and adaptability to current situations are essential to adequately address diverse challenges [1]. In this context, organizations emphasize the need for adaptable employees to respond to changes and developments in the economy [2] while maintaining their work capabilities [3]. In academic discourse, the phenomenon characterized by dynamics and change in the market economy is referred to as VUCA or the VUCA world [4].

Originally coined in the 1990s within US military vernacular, “VUCA world” described the emerging complex global scenario. By the 2000s, the business sector had adopted the term [5]. Presently, it serves as a theoretical framework highlighting technological upheavals, including digital transformation, and global economic challenges from financial and economic crises, as well as major health crises like pandemics. The relevance of the VUCA concept, particularly highlighted during the COVID-19 pandemic, underlines the impact of technological shifts [4].

The core elements of VUCA are often encapsulated by the terms “change” and “dynamism” [6]. The acronym breaks down into volatility, defined by fluctuations from the norm; uncertainty, stemming from insufficient information and unclear cause-and-effect relationships; complexity, where problems are too numerous to be managed individually; and ambiguity, characterized by unclear interpretations and ambiguous statements. While these constructs are primarily viewed negatively, they also present opportunities for growth and advantage, albeit more indirectly and infrequently. Moreover, there are significant overlaps among these constructs [4].

Organizations are increasingly challenged by persistent changes, particularly those intensified by digitalization, which demand swift organizational adaptations. Strategic management is compelled to respond to these new circumstances by adjusting organizations to cope with the loss of routine work processes [7]. Additionally, global economic developments and advancements in information and telecommunications technology since the COVID-19 pandemic have transformed the business environment. Factors like flexible work hours and demographic shifts add further complexity and demand flexibility [8,9,10]. Consequently, these dynamics require employees to be highly adaptable in terms of workplace design and health considerations [10,11].

It is crucial for organizations to acknowledge the impact of these dynamic external factors on their workforce [1]. An increasing gap in employee technological proficiency has emerged [12], leading to a scarcity of resources necessary to meet work demands effectively [13]. This deficit may diminish employee commitment to development and adaptation, posing various organizational challenges [14].

Under these conditions, it becomes imperative to explore how these stressors impact the workforce throughout Europe. An analysis of current stress data will provide a detailed picture of the burdens faced by workers, paving the way for an in-depth examination of psychosocial risks and health in the next section.

### 1.1. Psychosocial Risk and Health

Work incapacities related to stress are often classified under “mental disorders” and are further elucidated by ”psychosocial risks”. A 2022 EU report on workplace safety and health surveyed all 27 Member States of the European Union, as well as Iceland and Norway, to examine the post-pandemic landscape further. The study reveals that the predominant cause of work-related stress, identified by 46% of respondents, is intense time pressure or overload. Additionally, 26% reported problems due to inadequate communication or cooperation within their organization, while 18% identified a lack of autonomy or influence over their work pace and processes as stressors [15]. These issues are paralleled by physical health risks. For instance, ”severe eye strain” has been a significant concern from 2013 to 2020, with indications that it continues to rise [16].

Across the EU, half of the respondents report that digital technologies set the pace of work, and 30% believe that technology has augmented their workload [15]. The data highlight the growing physical and psychological strains facing white-collar workers, exacerbated by digital workplace dynamics. Further insights from absenteeism reports in Austria and Germany underscore the rising psychological stress. In Austria, absences attributed to psychological and behavioral disorders increased by about 20% from 2017 to 2021 [17,18]. In Germany, specifically, absences related to burnout rose by approximately 10% during the same period [19].

These trends indicate that digital work methods are intensifying employee stress and introducing new stressors. Changes in the work environment are increasingly harmful to workers’ health [20].

### 1.2. Theoretical Background

This shift from physical to psychological work demands, a transformation extensively documented in the current literature, has been evident since before the turn of the millennium. The field, occupational psychology, dedicated to enhancing workplace well-being and performance, has recognized these changes [21]. Bakker et al. [1] emphasize that companies require adapted leadership and development approaches that not only preserve but also promote the physical and psychological well-being of employees. This further suggests that, considering the stress data, employees suffer from a resource deficiency. We argue that updating work resources is necessary to enable a work design that is adapted to the situation [1].

A closer examination of the evolution of models in occupational psychology underscores the relevance of a progressively deepened understanding of the complex dynamics between workers and their environments.

Initially, models like the “Person–Environment Fit” [22] and the ”ASA-Model” (Attraction Selection Attrition Model) [23] focused on the alignment between employees and their work settings, highlighting the importance of a positive match for well-being [22,23]. These frameworks, together with subsequent studies [24,25], emphasized the compatibility of employees with their job environments, suggesting that well-being hinges on this alignment.

The Effort–Reward Imbalance (ERI) model introduced a different perspective by concentrating on the social exchange aspects of work, proposing that work-related stress should be balanced by rewards like salary or recognition to prevent adverse health outcomes [26]. Meanwhile, the Job Demands–Control (JDC) model explored the implications of job demands more deeply [27]. Addressing specific phenomena such as burnout, the “Maslach Burnout Inventory” (MBI) primarily tackled workplace exhaustion [28].

However, a methodological limitation of these earlier models was their unidimensional approach, which tended to capture only negative or positive elements of workplace phenomena [13]. To broaden the evaluation spectrum, the “Oldenburg Burnout Inventory” (OLBI) was developed to assess both positive and negative responses to workplace stress more comprehensively [1,13]. Despite these advancements, the specificity and limited applicability of common burnout models remained a challenge [29].

The variety of models highlights the intricate task of understanding and shaping effective work conditions. These frameworks are essential for proactively managing workplace stressors and enhancing outcomes like job satisfaction and productivity. Given the varied and complex nature of these models, the need for an integrative approach in occupational psychology becomes evident. An effective framework should encompass the diversity and complexity of work conditions while also providing the flexibility needed to address industry-specific characteristics [30].

Such a model would not only capture the diverse demands and conditions across various work contexts but also offer adaptive strategies for optimizing work environments. In this context, the Job Demands–Resources (JD-R) model emerges as delignated [31]. It aligns with these needs by assessing the impact of job demands and resources on employee well-being. It emphasizes the importance of both objective job characteristics and employee perceptions of these characteristics [32]. Additionally, it offers a broader and more universal perspective [1].

The development of the JD-R model has led to major advancements in understanding the interplay between job demands and resources, as evidenced by extensive research. Initially, the model validated the categories of demands and resources across three occupational groups, challenging the traditional notion that burnout is confined to person-oriented professions [28]. This research established that burnout is equally pertinent in professions primarily involving objects and data, leading to the identification of major categories of job demands and resources that apply across industries [1,13].

Subsequent studies have demonstrated that job demands are primarily linked to exhaustion and health issues, while job resources tend to boost engagement and organizational commitment [31]. Research has also demonstrated that high work engagement and adequate resources can enhance performance under challenging conditions without compromising well-being [32]. Further research has validated the model, illustrating, for example, that job demands can mitigate stress and vice versa [33]. Additionally, the motivational impact of job resources is notably strong when employees actively seek support [34].

Subsequent validation processes have expanded the JD-R model, demonstrating that personal resources positively affect work engagement similarly to job resources.

The purpose of this expansion is to give employees more control over their environment, effectively reducing job demands and increasing job resources [1].

The model underwent a significant evolution in the early 2000s when proactive elements like feedback, autonomy, and social support were integrated [33]. Compared to the initial version [13], this extension places a stronger emphasis on fulfilling the needs of employees, enabling them to perform their tasks with greater engagement [32]. There is also evidence suggesting that applying the JD-R model and implementing corresponding measures can not only improve the physical and psychological health of employees but also lead to cost savings [1].

Recent extensions of the JD-R model have updated it to reflect the current economic market conditions, and the complexities associated with a VUCA world. In such dynamic environments, leaders increasingly rely on the adaptability of employees through a process known as job crafting [9]. This process involves delegating responsibilities to enhance self-efficacy and motivation, thereby promoting well-being and increasing organizational effectiveness. Job crafting has been conceptualized as a dimension that influences job characteristics, thus establishing a connection with and extension to the JD-R model [35]. However, job crafting can also be seen as a response to the interactions between job demands and job resources [10,35].

Job crafting is generally seen as a bottom-up redesign process, where employees independently adjust their job characteristics by modifying the demands and resources of their work [9,29,36]. The objective is to ensure an optimal fit between the employee and the job [37,38]. However, not all self-initiated job adjustments result in positive outcomes. Some patterns, referred to as self-undermining, can be stressful and create obstacles that may lead to a self-reinforcing additional burden and potentially result in burnout [39].

In a VUCA environment, contraction-oriented job crafting aims to reduce complexity and contacts in daily tasks [9], while team-level job crafting involves both individual [40] and collaborative adjustments [41] to work tasks and relationships to achieve common goals. These adjustments can enhance performance at both the individual and team levels in dynamic environments. Furthermore, job crafting can coexist with top-down approaches to managing the complexity of current work demands and meeting employee needs [9].

Leadership is pivotal within the JD-R framework, exerting notable influence across several domains [42]. It directly affects job demands, job resources, and personal resources [43]. Leadership also acts as a moderator in the relationships between work and personal resources and motivation, and between job demands and stress [44]. Additionally, it impacts job design and the self-undermining behavior of employees [29].

**Figure 1:**
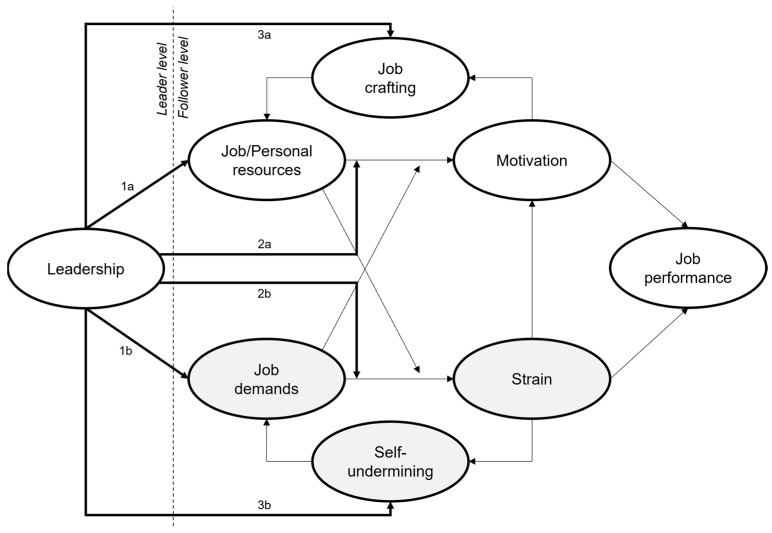
JD-R model expanded to include job crafting in reference to Tims, M. et al. [41] (own illustration).

Figure 1 illustrates how leadership impacts the JD-R model by directly affecting job demands and resources, moderating relationships between these elements and stress, and shaping working conditions that influence self-undermining behaviors [29,43,44].

Leadership is regarded as an emotional social resource, which, along with personnel practices as instrumental social resources, plays a vital role [45]. While instrumental resources provide the necessary conditions for adaptability through measures like knowledge transfer [46], emotional resources offer mental support and feedback [2]. Collectively, these can be regarded as management support [45].

For effective job crafting, it is essential that employees have a clear understanding of and identification with their roles, allowing them to adjust their tasks and responsibilities in alignment with both personal and organizational goals. This approach minimizes the negative impacts and additional burdens of job crafting [47]. Coordination with organizational support measures is critical [9], and it is vital for leadership to understand and provide the necessary support [48]. Leadership behaviors, as well as work and personality traits, are seen in the literature as initiators for job crafting [48].

Overall, job crafting extends the JD-R model to address the dynamic changes in the VUCA market economy. It is becoming necessary when deviations from usual routines create unmet job needs that require adjustments for employee agility and action capacity [4]. The role of leadership and employee interactions with it are crucial. The effectiveness of job crafting depends on specific support functions, which must be carefully considered [45,46].

### 1.3. Research Gaps and Aim of the Study

The theoretical specification of the JD-R model in response to the challenges of a VUCA world, particularly through job crafting, has revealed several research gaps that became apparent during the research:

1. JD-R research should take causal inferences more seriously and further investigate cause-and-effect relationships using appropriate methods [42]. Future research could detail how employee outcomes are simultaneously influenced by multiple job characteristics [49].
2. Bakker et al. [1] suggest that methods allowing a deeper, multilayered examination of organizational and psychological phenomena utilizing the JD-R theory would be beneficial. This includes exploring how different variables (state and trait) interact within a model and their impacts on the work environment.
3. There is a need for advanced methods to identify stressors not captured by the JD-R model, aiming to enhance its applicability and accuracy in assessing work stress [50].
4. The impact of digitalization on work demands and resources across various work environments and industries should be meticulously examined to gain a more comprehensive understanding. Continuous adaptation of the JD-R model to rapidly changing digital work environments is required [51].
5. Future research should aim to include a diversity of cultural frameworks, such as individualistic cultures, along with diverse geographic locations and sectors, to validate and expand the applicability of findings on job perceptions and proactive employee behavior. Addressing this deficiency can provide deeper insights into employees’ personal experiences and perceptions. Overall, there is a notable gap in qualitative research on this topic. Such studies will enhance our understanding of how cultural factors and management practices like job crafting impact employee engagement [45].

Our study primarily focuses on identifying and investigating the third gap within the JD-R model, which involves recognizing currently unnoticed stress and strain factors that could expand the model [50]. This focus is crucial for enhancing understanding and applicability across diverse work settings, particularly in changing environments [42]. To capture the full meanings and contexts of constructs at the individual, group, and organizational levels, we utilize qualitative research approaches, which allow us to understand the nuanced dynamics within these settings [52,53,54]. Given the scope and context of our study, we also briefly explore the fourth and fifth gaps, which are intrinsically linked to our primary focus on the third gap. This includes exploring individualistic cultures and ensuring a deeper understanding of job crafting mechanisms and the impacts of digital transformation [45,50,51].

## 2. Materials and Methods

This research employs qualitative case studies using semi-structured interviews to systematically gather data [54]. The study utilizes a comparative analysis of two fundamentally different companies to identify common characteristics that extend beyond individual corporate contexts. This approach seeks to enhance the external validity of the findings, ensuring their applicability across diverse settings. A specific focus is placed on organizations affected by technological change and operating predominantly in office-oriented (“white-collar”) work environments due to their relevance to the examination of stress data.

To ensure the empirical data’s quality, we guided participant selection within the organizations through a multilayered process. Initial exploratory discussions with the organizations identified key touchpoints relevant to the research theme. These discussions facilitated the identification of specific characteristics for potential interviewees, aligning strongly with the research questions. This methodical approach ensured an objective and focused selection of participants. Following this, the research team initiated contact and began data collection through semi-structured interviews. The interviews were based on the “JD-R model” and Probst’s Model of “Knowledge Building Blocks” [55]. Flexibility was maintained in the interview guide to accommodate the individual experiences and perspectives of participants. Data collection continued, with interviews being conducted and analyzed, until theoretical saturation was reached.

Enhancing the study’s reliability involved conducting a preparatory test that refined the interview methodology based on received feedback. Participants received informational materials detailing the research background, interview procedures, data protection, and usage protocols well in advance.

In the first case study, 14 members of an emergency rescue organization covering a broad range of roles were interviewed. In the second case study, seven employees from an industrial organization representing various hierarchical levels were interviewed. The differing number of participants in each study was due to the data collection approach, which continued until no additional theoretically relevant information could be gleaned. To preserve the authenticity of the theses and analytical results in Section 3, the original German statements were carefully translated into English by the authors, ensuring minimal bias.

GABEK^®^ (Ganzheitliche Bewältigung von Komplexität), developed by Josef Zelger and supported by WinRelan software, was selected and applied as the evaluation method. GABEK^®^ is distinct from other qualitative content analysis methods in its ability to handle large datasets without reduction, allowing for deep insights into relationships. Consequently, it serves as a structured approach for analyzing and systematizing data in variable and complex organizational contexts. Theoretically, GABEK^®^ is based on the concept of perceptual “Gestalten” (“Wahrnehmungsgestalten”) by Carl Stumpf. GABEK^®^ uses the sentence as the minimal meaningful element [56,57]. This premise underpins the capture of complex organizational meanings through sentence analysis, visualizing them as undirected graphs where key terms form nodes linked by participant statements. This methodology facilitates the mapping and aggregation of subjective meanings into a comprehensive network, which helps reveal inter-subjectively used meaning structures and clarify terms [58].

GABEK^®^ offers different, specifically utilizable levels of evaluation that can vary in the desired level of detail. In this scientific case, a stepwise evaluation beginning with the “Gestaltenbaum” (“structure tree”) proved effective. The structure tree serves as a synthesis of the most relevant problems and challenges of the examined organizations. This structure tree visualizes the mental space over a specific research area, offering an overview and uncovering the most relevant thematic areas. Organized on three levels, it provides a broad content overview at the highest levels (Hyper-Hypergestalten), which allows for the deduction of decision-making areas and overarching focal points for leadership. These are linked on the underlying levels, providing detailed insights through employee goals and measures.

This framework aids in refining and expanding contextual understanding by decomposing complex data into smaller, conceptually framed postulates with defined underlying meanings, making them processable by the human brain. This initial evaluation step checks data alignment with the problem statement and acts as a validation step for the detailed analysis that follows. Insights from lower levels of the tree can also identify measures to address problems described at higher levels [58].

Following the Gestaltanalysis, specific terms and network analyses further deepen insights into the potential mechanisms of action that can be derived in accordance with the research gap. These analyses aim to examine the themes or terms extracted from the Gestaltanalysis more closely, using influential terms in proximity to a core term and analyzing their relationships. For a more comprehensive ontology analysis, the entire network is included and simplified into a linguistic network of reduced complexity according to specific rules, enabling deeper insights into the organizational members’ perspectives [57,58].

## 3. Results

**Figure 2:**
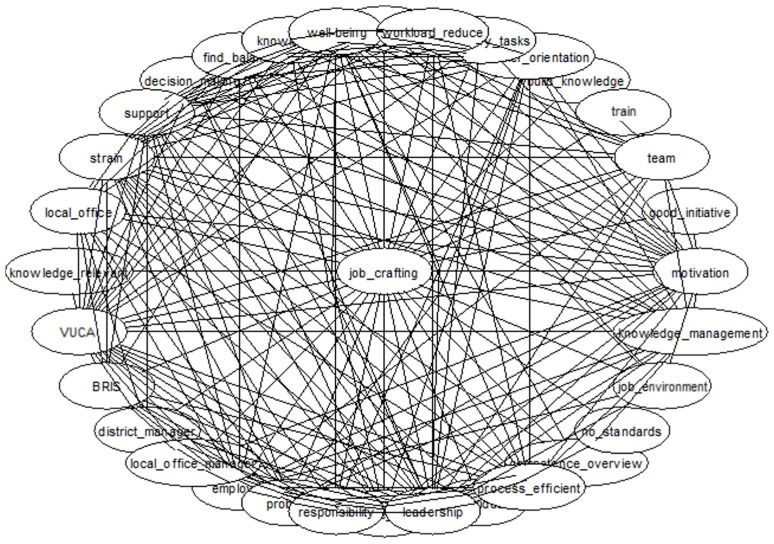
Data complexity illustration network (own illustration).

The current understanding and state of research on the JD-R model provides a structured framework for its exploratory development. Furthermore, the application of GABEK^®^ within the VUCA world context enables a detailed identification and investigation of additional phenomenal influences on demands and resources. In response to this complex and dynamic setting, Figure 2 intends to illustrate the complexity and intricate interconnections of the collected data.

### 3.1. Gestaltanalysis

**Figure 3:**
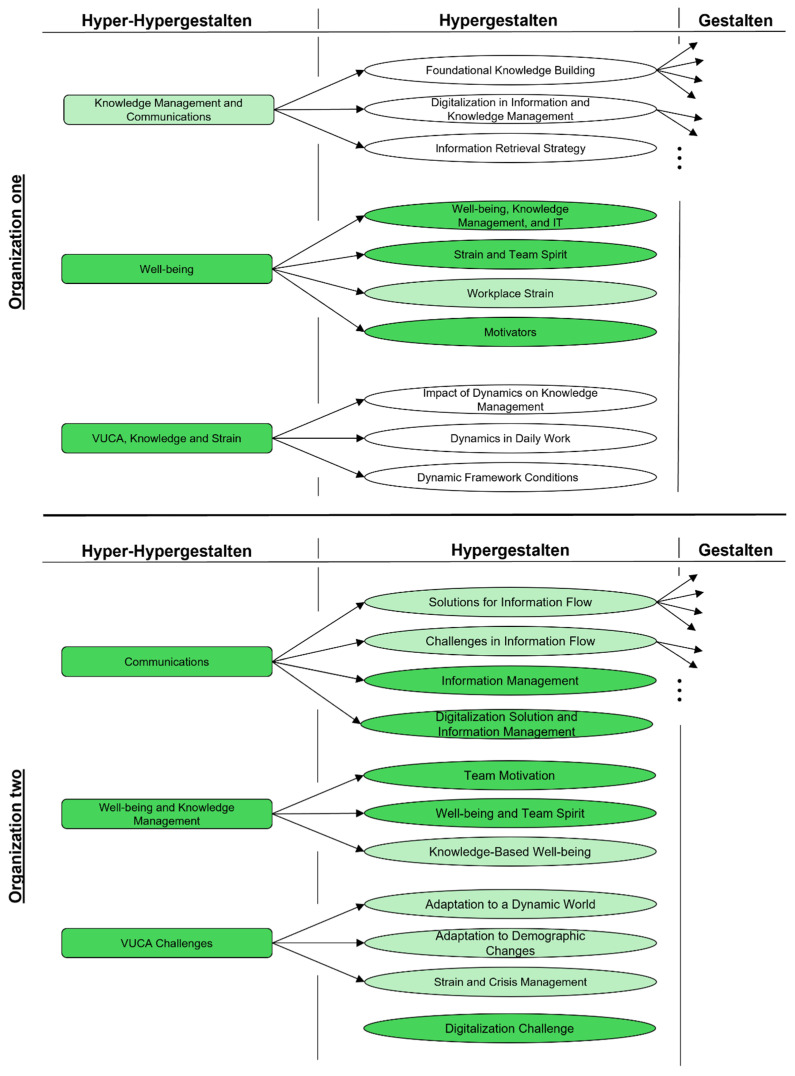
Structure tree of the analyzed organizations (own illustration).

The initial step in our analysis involved creating an overview of the data by forming linguistic Gestalten. At the Hyper-Hypergestalt level, common challenges emerge across both organizations studied. Figure 3 represents a simplified version of the coded structure tree, with color-coded highlights of straining constructs, illustrating the identified thematic areas organized according to their origin and impact. Initial data suggested that processes of “digital transformation” and “changing work demands” pose significant challenges in “communication” and “knowledge management”, which can lead to “strain”. A comparative analysis of the underlying texts was conducted to gain deeper insights.

The analysis highlights the growing complexity in the workplace, driven by rapid changes in the digital landscape and dynamic work tasks. A prominent issue identified is the inconsistent use of multiple IT systems across the organizations, compounded by poor communication and inconsistent directives. This inconsistency presents particular challenges for less tech-savvy employees, leading to difficulties in managing and utilizing information within complex data structures. These issues significantly impact knowledge management, where noticeable struggles include the autonomous execution of tasks and the efficient updating and management of critical job knowledge. Additionally, communication gaps hinder smooth adaptation to new technologies and ongoing digitalization, affecting the balance between personal interaction and the use of digital communication tools. At the Hyper-Hypergestalt level, the work environment is characterized by increasing complexity, influenced by rapid digital changes and exacerbated by communication issues at the organizational decision-making level. This complexity necessitates strategic adjustments.

An in-depth analysis at the second and third Gestalt levels was conducted to bridge existing knowledge gaps and reveal mismanagement. This step provided deeper insights into underlying dimensions and aided in the development of targeted intervention strategies. The Hypergestalt level reveals a consensus on prevailing problem categories and a methodologically induced tendency towards specification. Interestingly, the initially problematic diversity of IT systems is now viewed more positively, particularly for their contribution to enhancing transparency and reducing strain. A rising demand for clear structure and transparent responsibilities indicates a shift towards optimizing organizational processes. This suggests a more systematic approach to addressing challenges in knowledge and communication management. Detailed, context-specific investigations reveal that organizations are actively pursuing tailored solutions to meet their unique challenges, reflecting a proactive approach to adapting to evolving workplace dynamics.

Initial findings from the structure tree indicate that dynamism and changes in the work context affect employee performance and well-being. It is evident that current management methods and structures often fail to keep pace with these rapid changes, potentially resulting in declines in both performance and motivation. Consequently, there is a trend towards the development and implementation of appropriate structures and support measures that effectively align monetary and health considerations with these evolving challenges.

To delve deeper into the data, the network analysis outlined in Section 2 was conducted. This analysis aimed to explore potential mechanisms of action for the identified topics, enhancing our understanding of how specific changes and interventions influence organizational dynamics and employee outcomes.

### 3.2. Network Analysis

Specific network graphs for key terms such as “job crafting”, “leadership”, and “self-undermining” were developed. The network analysis began with a focus on “leadership”, which serves as the pivotal point for job crafting in the model, as it serves as the pivotal point for job crafting within the model of Tims M et al. [41]. This initial focus provided a foundational understanding that informed the analysis of subsequent processes.

**Figure 4:**
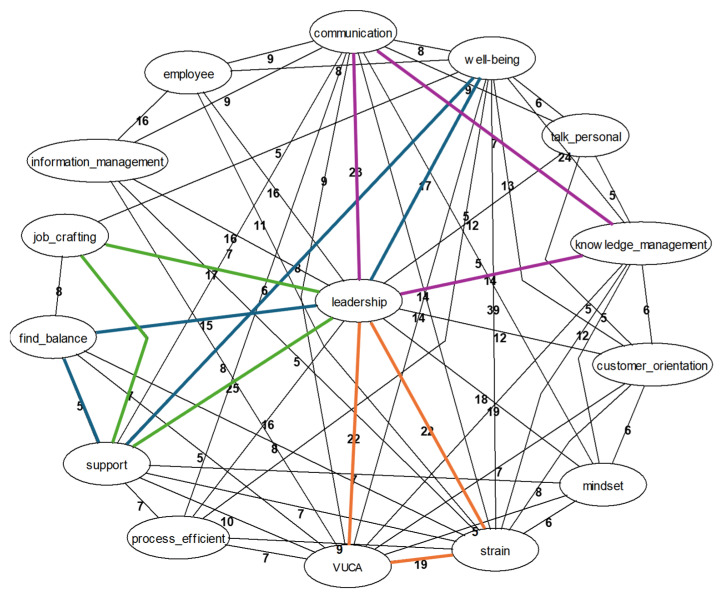
Network graphic: “leadership” (own illustration).

Figure 4 illustrates the complexity and interconnectedness of the data, using the key phrase “leadership”. Due to the intricate nature of the network, a specific focus was applied to ensure clarity and relevance in the investigation. This methodological approach was shaped by insights from the literature review and findings from the Gestaltanalysis, which guided the selection and emphasis of key areas within the graph. The examined sub-networks are visually highlighted in color, making them easily distinguishable within the overall network. The numerical values associated with the interconnecting lines of the key concepts within the linguistic network graph represent the count of sentences that provide detailed explanations of the relationships between these concepts.

In dynamic environments, leadership requires quick reactions and adaptability to changing conditions. Agile decision-making processes are crucial for navigating organizations through unpredictable challenges, underscoring the need for enhanced decision tools that facilitate quick and informed choices. One participant noted the following:

*“We need better decision-making capabilities to become faster and more efficient”.*

[Ai5]

The COVID-19 pandemic underscored the importance of knowledge management as a critical component of effective leadership. Leaders had to identify and communicate essential information without overwhelming employees. One participant shared their approach to communication during the pandemic:

*“I usually decide myself what to communicate outdoors. During the Corona times, we almost got daily updates from the (Anonymized) from the doctor, I passed on everything one-to-one”.*

[Bi8]

Additionally, another participant emphasized the challenge of information selection.

*“As department heads, we receive quite a lot of information from the leadership, and I always have to sort out what to pass on because you can’t overwhelm them…”*

[Bi6]

Adequate communication is crucial, especially when vital information is disseminated selectively through “Key users”, to ensure comprehensive engagement. This selective approach often fails to engage all stakeholders, thus impairing organizational effectiveness. Reports further suggest that existing systems are often misaligned with current challenges, partly due to an overabundance of software tools. Moreover, especially among older employees, there is resistance to changes, often rooted in long-established routines.

*“Problems with the system variety? Because I actually feel comfortable in my world. And so do my colleagues in their worlds, even if they file things a bit differently than I do, because if (unintelligible) my folder structure is, let’s say, set up country-specific, then customer-specific, and then depending on which company you go into”.*

[Om8]

Efficiently reducing secondary and competence-mismatched tasks, such as handling general customer inquiries and compiling experience reports, for knowledge carriers is crucial for optimizing resources. By implementing targeted controlling and support measures in these areas, organizations can enable employees to concentrate more on their core tasks.

Employee well-being and motivation are deeply connected to the effectiveness of leadership and the robustness of organizational support. Strain, which arises from mismatches between organizational demands and available resources, can undermine employee motivation. Proactive and supportive leadership is essential to foster a stable work environment, offering employees both security and appreciation.

*“Well-being? Yes, sure, if you don’t feel comfortable, then yes, very, very important. It’s not always about the money or anything else that keeps you in your job; your environment has to be right, your boss has to be right, you have to feel comfortable, and then you’ll also perform, then it works, it’s a mutual give and take, if all that fits, then yes, it works out”.*

[Oz9]

**Figure 5:**
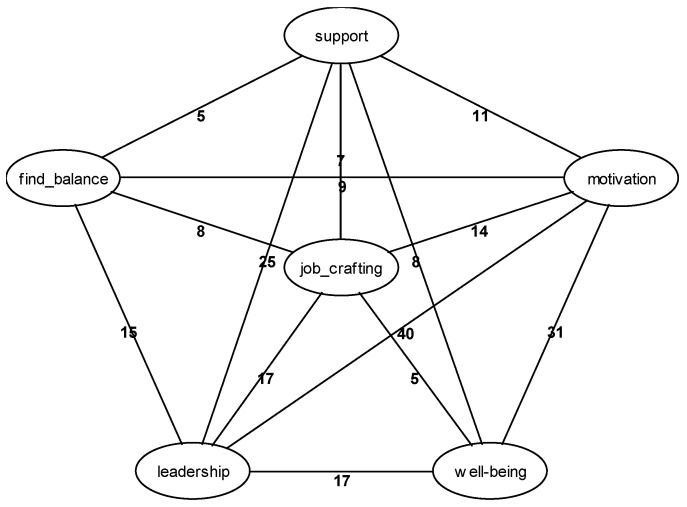
Network graphic: “job crafting” (own illustration).

In dynamic environments, effective leadership is crucial for maintaining a balanced flow of information, preventing overload and ensuring all employees are informed timely and comprehensively. By implementing improved communication strategies and efficient knowledge transfer, leaders minimize uncertainties and enable employees to align their performance with organizational demands. Simultaneously, structured, supportive, and adaptable leadership facilitates job crafting by allowing employees to tailor their work methods to the specific requirements of their tasks, thereby contributing to motivation. Synchronizing leadership and organizational requirements with available resources, and promoting structures that support job crafting and knowledge management, are pivotal in enhancing employee well-being and overall productivity. Next, we will explore deeper aspects of “job crafting”, as depicted in Figure 5.

The network analysis validates the existing literature on job crafting and extends this perspective with contextual insights. The in-depth analysis highlights the critical role of balanced leadership characteristics in enhancing leadership effectiveness. A participant underscores the value of effective leadership, stating the following:

*“Finding the middle ground […] and being a good listener is crucial”.*

[Ok5]

Challenges in job crafting often stem from the need to establish adequate support and resources. Effective leadership requires emotional intelligence and methodological expertise to appropriately address individual team members’ strain thresholds. Insufficient support and feedback can cause employees to feel organizationally and emotionally disconnected, leading to frustration:

*“I believe that we simply lack the right leader who is always present. Leadership that provides just the right amount of support would be extremely important”.*

[Dd3]

Another aspect of support is reflected in the feedback employees receive from successful projects and customer satisfaction. One participant notes the value of such feedback:

*“Because it’s direct confirmation for yourself [...] and the feedback from the customer, this praise, it often feels like something that no amount of money in the world can really compensate for”.*

[Od4]

This perspective becomes crucial amid a scarcity of internal resources and tailored support, potentially driving employees to seek motivation independently, sometimes defiantly. To effectively foster job crafting, leaders must ensure transparent communication and manage employee pressure adequately. It is crucial to boost efficiency by investing in further education and enhancing personnel resources. Leaders should empower teams to independently shape their work roles and offer psychosocial support as mentors.

*“More transparency, what are we doing, who is doing what, and what I said earlier, just the connection between full-time and volunteer staff, the main office must get it into their heads that they are there for the (Anonymized) offices, and that they are actually the ones who are the engine of the (Anonymized) service because without local offices there is no main office”.*

[Gf8]

**Figure 6:**
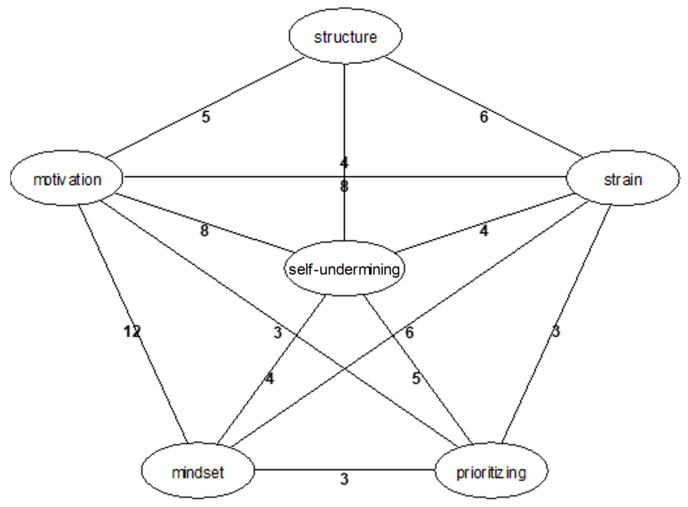
Network graphic: “self-undermining” (own illustration).

The pivotal role of leadership within job crafting is further emphasized. Leaders must harness emotional intelligence and perceive the demands of employees with balanced support to recognize and appropriately respond to the individual stress thresholds of team members. In this capacity, they serve not only as managers but also as mentors to their teams. The presence, approachability, and transparent communication of leaders are essential for effective stress management and fostering a supportive and positive culture. These elements are critical to fully leveraging the potential of job crafting in a context-based manner and optimizing the work environment. Next, we will explore deeper aspects of “self-undermining”, as depicted in Figure 6.

The network in-depth analysis further expands the self-undermining aspect of job crafting in dynamic work environments. Specifically, inadequate awareness of individual competencies can lead to inefficiency, stress, and self-undermining, as one participant described:

*“I struggle to effectively utilize my skills and those of the team, which unnecessarily creates problems. Despite bringing my entire education and professional experience, I don’t always manage to optimally use each team member’s specialized knowledge”.*

[Oe9]

Furthermore, poor task prioritization skills can result in time constraints, structural deficiencies, and overload, which further reinforce self-undermining behaviors. Another participant expressed frustration with task management:

*“I always try to prioritize tasks and constantly reprioritize when there are sub-tasks, but yeah, that often doesn’t work properly; I can only do so much work”.*

[Px3]

To counteract self-undermining, it is essential to establish structured processes and clear prioritizations. Organizations should cultivate an integrative mindset across all levels to leverage both individual and collective potentials effectively. Reflecting on a structured environment, a former bank employee shared a positive experience:

*“At the bank, everything was very structured, although each day was different, but very structured. At first, I needed some time because we have so little structure that I had to find a structure for myself that made things easier for me”.*

[Ac3]

Such structured environments enable employees to establish clear personal workflows, thereby enhancing efficiency.

A central strategy to mitigate self-undermining is to foster structured processes, mindset initiatives, and heuristics. These strategies enhance motivation and reduce the internal conflicts and self-doubt that arise from inconsistencies and indulgences. A solid understanding of the purpose and significance of work fosters a goal-oriented mindset that aligns with both the organization’s vision and mission as well as the personal ambitions of employees. Overcoming internal barriers and dismantling silo thinking is crucial to improve collaboration and fully utilize collective potential. By strategically implementing these elements, organizations can secure sustainable competitive advantages and guard against self-sabotaging behaviors among employees.

### 3.3. Synthesis of Key Findings

Reflecting our research focus, this study aims to uncover previously unrecognized demands and resources, thereby deepening and expanding the JD-R model through job crafting. Additionally, it situates the dimension of leadership within a framework pertinent to our study.

Our results primarily demonstrate through Gestaltanalysis how digital transformations and evolving work demands affect the dimensions of knowledge management and communication, which, in turn, negatively impact employee well-being.

It also became clear that specific subordinate phenomena, particularly inconsistent implementation and specification of IT systems, coupled with communication deficits, act as major drivers of work environment complexity. This underscores the urgent need for improved structures, communication strategies, and competence-building measures tailored to specific contexts.

Furthermore, the findings elucidate mechanisms within the research context, indicating that changes in complex occupational environments necessitate a solid knowledge base combined with the freedom to utilize it effectively. Efficient management, competence development measures, and access to information are critical for building and providing this knowledge. These efforts aim to reduce stress caused by disrupted routines and subsequent declines in time management, which ultimately negatively impact employee well-being.

Leaders play a crucial strategic role, as highlighted in our analysis, by leveraging emotional intelligence and setting clear goals and priorities through proactive and structured approaches. These efforts strengthen organizational attachment by mitigating the risks posed by newly emerging demands. Adaptive leadership and structured support in developing employee heuristics are therefore essential for promoting successful job crafting and effectively countering self-undermining behaviors, thereby unlocking potential performance resources.

In the rapidly evolving digital era, comprehensive organizational adjustments and more proactive employee leadership are indispensable to enhance workforce performance and well-being. Job crafting, in conjunction with adapted leadership strategies, is crucial for timely knowledge provision and minimizing self-undermining actions. These strategic initiatives effectively address challenges related to time and information shortages, thereby unlocking full employee potential when considering current demand effects.

## 4. Discussion

Current findings highlight the influence of leadership characteristics on employee well-being and emphasize the importance of a work environment that fosters adaptability and flexibility. Leaders act as a central force, orchestrating working conditions, resource management, and employee motivation to support their demand adaptability and well-being [29,31,43,44,45]. Our research has refined leadership requirements by identifying the evolving needs of employees in a constantly changing work environment. Against this backdrop, a deeper engagement with leadership in the context of well-being is encouraged to integrate the empirical findings into the existing literature.

A literature review underscores the crucial role of leadership in employee well-being, especially in a world of continual change. Leadership is instrumental in enabling effective responses to complex challenges by harnessing collective competencies and knowledge [59,60,61]. Approaches promoting an open exchange of ideas and knowledge among professionals have shown positive effects, such as improved care quality and employee well-being in healthcare settings [60]. Similarly, leadership that involves employees in decision-making promotes innovative behaviors through a culture of knowledge sharing [62]. Furthermore, leadership that values diversity and acknowledges individual contributions correlates with enhanced workplace well-being in public organizations [63]. These findings demonstrate how employee-centered leadership is essential in creating a positive work environment and enhancing overall well-being [52].

However, the literature reveals that the impacts of certain leadership characteristics on well-being can vary, highlighting the need for further investigation into these outcomes. This research should focus on identifying the specific conditions under which leadership approaches can be most effective [52]. Additionally, understanding the necessary personality traits of leaders is crucial to ensure compatibility with their leadership requirements, which is fundamental for facilitating effective job crafting. A lack of comprehensive understanding of these traits can impede the development and implementation of job crafting initiatives [64].

The scientific literature frequently highlights in this context the significance of leadership in adapting to changes, accounting for a significant share of the research in this domain [52]. This focus is reflected in our findings, especially concerning the adaptive abilities of leaders. Our data emphasize that in changing environments, the adaptability of leaders is crucial. An expansion of leadership competencies related to adaptation and resource conservation is evident. These skills are essential for swiftly responding to changes that could potentially increase stress, as well as for adapting to ongoing transformations. It is also important to ensure the optimal utilization and distribution of resources such as time or workforce to mitigate stress and uncertainty among employees.

Leaders who facilitate access to necessary information help employees make informed decisions, conserve resources, and reduce stress caused by uncertainty and inadequate decision-making. However, the methods of information and knowledge transfer must be carefully managed to prevent information overload while ensuring that relevant information is strategically conveyed. Leaders need to demonstrate adaptability skills to achieve the optimal balance between information flow and filtration, which positively impacts employee well-being. This balance is critical for creating an environment where employees can perform effectively, unburdened by excessive data or a lack of necessary insights. Nevertheless, it is crucial to acknowledge the limitations of these required capabilities. The culture of traditionally structured organizations often falls short of the demands for contemporary and effective leadership practices [65]. Our findings extend this view by illustrating how such leadership can adeptly respond to the challenges of a dynamic world. However, adaptability should not only be personal to the leaders but should also be reflected in the entire organizational structure, considering the ability to integrate dynamic capabilities [66]. This highlights the necessity for comprehensive adaptability that encompasses both personal leadership behaviors and organizational practices. Nevertheless, without cultural and structural changes, implementing positive leadership may prove challenging. This insight lays the groundwork for further empirical research and theoretical development on the relationship between job crafting and leadership dynamics.

### 4.1. Practical Implications

It is recommended that leaders be flexible, empathetic, and open to learning, making decisions based on current and relevant information and continuously adapting their strategies to encompass a broad perspective. A robust knowledge management system and strong commitment are essential to prevent information overload and ensure that employees receive the necessary support, thus reducing stress and enhancing well-being. Furthermore, a corporate culture that encourages knowledge exchange plays a key role in fostering a strong, resource-oriented work environment. Clearly, integrating knowledge into leadership processes is critical for creating positive work environments and improving employee well-being and retention.

### 4.2. Limitation

The primary limitation of this research stems from the constrained sample size of the organizations examined. This limitation could potentially limit the transferability of the results to other sectors. To mitigate this limitation, we implemented measures such as attaining theoretical saturation, focusing on contrasting cases through theoretical replication, and concentrating on groups vulnerable to stress. These approaches enhance both the robustness and the validity of the findings, despite the limited sample size. Moreover, the research design involves partially directed participant selection, which could introduce subjective biases. Despite these limitations, the study was conducted with strict adherence to the principles of good scientific practice.

### 4.3. Future Research

Further research is crucial to assess the effectiveness of leadership strategies thoroughly and refine intervention methods. Future studies should explore the performance of different leadership models across various organizational contexts, identifying conditions under which they are most effective. It is also vital to examine how these strategies adapt to diverse cultural and operational contexts to enhance workplace well-being and organizational efficiency [60].

The stress data, collected on a Europe-wide scale for the theory introduction, reveal a significant research gap. Approximately one in six individuals across all EU countries report suffering from stress factors that are not captured in the studied categories [15]. This phenomenon presents a promising research opportunity to explore the nature and effects of these unclassified stress factors more precisely.

## 5. Conclusions

This study demonstrates how white-collar employees in organizations can actively shape their work conditions through job crafting, optimizing resource use and managing work demands effectively. Findings confirm that the JD-R model, expanded to include job crafting, offers a strategic framework for addressing the challenges of digital transformation and dynamic work environments. The results highlight the need for adaptable leadership strategies that foster an environment conducive to job crafting, thereby enhancing effectiveness and employee well-being.

Highlighting the indispensable role of leadership in the face of organizational change, this study identifies critical phenomena and mechanisms in dynamic environments that are essential for aligning employee well-being with the strategic aims of the organization. It is essential for leaders to exhibit emotional intelligence, adaptability, and knowledge relevance awareness. An effective knowledge management system is pivotal to avoiding information overload and ensuring that employees receive the necessary support, thereby reducing stress and promoting well-being. Additionally, a corporate mindset that supports knowledge exchange contributes to the development of a strong, resource-oriented work environment. By adapting their decisions to current information and effectively imparting these principles to employees, leaders can reduce stress and enhance well-being. Providing foundational insights for further research, this study underscores the critical need to rigorously test the empirical findings through quantitative methodologies across diverse sectors and settings to assess their relevance and reliability.

## Data Availability

The data presented in this study are available on request from the first author. The data are not publicly available due to privacy restrictions.
